# Modifiers of radiation effects on breast cancer incidence revealed by a reanalysis of archival data of rat experiments

**DOI:** 10.1093/jrr/rrac090

**Published:** 2023-01-07

**Authors:** Tatsuhiko Imaoka, Mayumi Nishimura, Kazuhiro Daino, Shizuko Kakinuma

**Affiliations:** Department of Radiation Effects Research, National Institute of Radiological Sciences, National Institutes for Quantum Science and Technology, Chiba 263-8555, Japan; Department of Radiation Effects Research, National Institute of Radiological Sciences, National Institutes for Quantum Science and Technology, Chiba 263-8555, Japan; Department of Radiation Effects Research, National Institute of Radiological Sciences, National Institutes for Quantum Science and Technology, Chiba 263-8555, Japan; Department of Radiation Effects Research, National Institute of Radiological Sciences, National Institutes for Quantum Science and Technology, Chiba 263-8555, Japan

**Keywords:** animal experiment, effect modifiers, individual response, interaction, risk model, data archive

## Abstract

Cancer risk after exposure to ionizing radiation can vary between individuals and populations, but the impact of factors governing those variations is not well understood. We previously conducted a series of carcinogenesis experiments using a rat model of breast cancer, in which 1654 rats born in 2002–2012 were exposed to γ rays at various doses and ages with or without non-radiation factors including high-fat diet, parity and chemical carcinogens. We herein reanalyze the incidence data from these archival experiments to clarify the effect of age at exposure, attained age, radiation dose and non-radiation factors (i.e. fat, parity, chemicals and birth cohorts) on radiation-related mammary cancer incidence. The analysis used excess relative risk (ERR) and excess absolute risk (EAR) models as well as generalized interaction models. Age-at-exposure dependence displayed a peak of susceptibility at puberty in both the ERR and EAR models. Attained age decreased ERR and increased EAR per unit radiation dose. The dose response was concordant with a linear model. Dietary fat exhibited a supra-multiplicative interaction, chemicals represented a multiplicative interaction, and parity and birth cohorts displayed interactions that did not significantly depart from additivity or multiplicativity. Treated as one entity, the four non-radiation factors gave a multiplicative interaction, but separation of the four factors significantly improved the fit of the model. Thus, the present study supports age and dose dependence observed in epidemiology, indicates heterogenous interactions between radiation and various non-radiation factors, and suggests the potential use of more flexible interaction modeling in radiological protection.

## INTRODUCTION

Cancer risk after exposure to ionizing radiation can vary between individuals and between populations, but the factors that govern such variations are not well understood [1]. Risk of radiation-related cancer is generally described by excess relative risk (ERR) and excess absolute risk (EAR) models. In ERR models, the disease rate is expressed as a product of the baseline risk (i.e. risk in the unexposed population) and a relative risk that is a function of radiation dose. In the EAR model, the rate is formulated as a sum of baseline and radiation-related risks. Both baseline and radiation-related risks are subject to modifications by environmental, lifestyle and genetic factors that are characteristic to individuals and populations as well as the age of the subjects at risk and the age at exposure. The ERR model assumes a multiplicative interaction between radiation and the modifiers of the baseline risk, whereas the EAR model assumes an additive interaction. These risk models are representative of a spectrum of interactions that can, more realistically, range from antagonism, through sub-additivity, additivity and multiplicativity, to supra-multiplicativity.

Breast is one of the organs most susceptible to radiation-related carcinogenesis, yet the risk of breast cancer is also influenced by diverse lifestyle and environmental factors [2, 3]. The diversity in lifestyles and environments among populations often complicates the analysis of radiation-related cancer risk of many tissues including breast cancer. For example, Japanese and US populations have different baseline risks of breast cancer [4]. In a pooled analysis of multiple cohorts, the most preferred ERR model suggests different dose-related increases in ERR between Japanese and US populations, whereas the best EAR model infers that the extent of the increase in EAR per unit dose does not differ among populations [5]. The current radiological protection system therefore adopts the EAR model for risk transfer (i.e. prediction of radiation-related risk in non-Japanese populations based on the Japanese atomic bomb survivor data) for breast [6]. On the other hand, the breast cancer incidence has increased over time in the Japanese population itself. As a result, in the studies of the Life Span Study (LSS) cohort of Japanese atomic bomb survivors, the ERR model suggests an identical dose-related increase among different birth cohorts, whereas the EAR model indicates different dose-related increases among the birth cohorts [2, 7]. The birth cohort and the age at exposure are equivalent in the LSS cohort, as the exposure occurred exclusively in the year 1945. Importantly, in both the pooled analysis [5] and the LSS reports [2, 7], the ERR model suggests no significant impact of the age at exposure on radiation-related breast cancer risk, whereas the EAR model indicates a significant effect of the age at exposure. As such, the choice between these risk models greatly complicates the inference regarding application to radiological protection.

Animal experiments provide important information regarding the biological effects of radiation, and they complement epidemiological studies. Reanalysis of archival animal data can produce new important information required in many fields including radiological protection [8–11]. To date, many animal studies have addressed the interaction of radiation with various non-radiation factors [1]. Nevertheless, the interaction in these animal studies has only rarely been assessed quantitatively [12, 13]. We have conducted a series of carcinogenesis experiments using a rat model of breast cancer, in which rats were exposed to ^137^Cs γ rays and subjected to other non-radiation factors including a high-fat diet, parity (i.e. history of childbirth) and exposure to carcinogenic chemicals [14–21]. One of the rat experiments indicated a significant variation in the baseline cancer risk related to the birth cohort [22]. Thus, these previous data provide an opportunity to test the interaction between radiation dose and various non-radiation factors. As mentioned above, age is also an important modifier of radiation effects. Whereas many animal studies have focused on the influence of age at exposure [1], the effect of attained age has not been as rigorously studied. In our previous studies on rat mammary carcinogenesis [14–22], the weekly palpation data collected were connected with the pathological diagnosis upon autopsy, and thus this information provides an opportunity to retrospectively analyze the influence of attained age in those experiments.

The present study reanalyzes the pooled incidence data from these past experiments (consisting of 1654 rats in total) to quantify the interaction of the effects of high-fat diet, parity, chemicals and birth cohort with radiation. The study also evaluates the modification by age at exposure and attained age as well as the shape of the dose response. The results not only support the trends of age and dose dependence recently suggested by epidemiology but also suggest significant heterogeneity in the modification of radiation-associated risk by non-radiation factors.

## MATERIALS AND METHODS

### Animal experiments

The data sets from previous experiments are shown in Table 1. In these experiments, female Sprague–Dawley rats (Jcl:SD, CLEA Japan, Tokyo, Japan) were fed with a standard diet (CE-2, CLEA Japan), palpated weekly, and autopsied upon general health deterioration, natural death or predetermined termination endpoints for pathological diagnosis of the palpable mammary tumors [14–22]. Termination occurred at 90 weeks of age [18, 20, 22], 100 weeks of age [21] or was not set (i.e. the rats died naturally or were sacrificed only upon general deterioration) [19]; in a subset of studies, the rats with one or more palpable tumors were autopsied at 50 weeks of age, and those without palpable tumors at that time were checked until a tumor(s) could be palpated, at which time autopsy was done [14–17]. Acute whole-body irradiation with ^137^Cs γ rays (0.5–0.6 Gy/min) was performed once at either 1, 3, 7, 13 or 15 weeks of age. In some experiments, the rats were fed a high-fat diet (23.5% corn oil in AIN-76A, CLEA Japan) from 9 weeks of age [14–17], which increased the body weight by 12%. In other experiments, they were mated with male Jcl:SD rats and allowed to deliver and nurse a litter [21]. The postpubertally irradiated groups were excluded as this previous study did not identify any modifying effect of parity in those groups [21]. To evaluate the influence of chemical carcinogen exposure, the rats were intraperitoneally injected with 1-methy-1-nitrosourea (MNU) at 20 mg/kg at 3 or 7 weeks of age (denoted MNU20/3 and MNU20/7, respectively) or at 40 mg/kg at 7 weeks of age (denoted MNU40/7), or they were administered 2-amino-1-methyl-6-phenylimidazo[4,5-b]pyridine (PhIP, 40 mg/kg/day) daily for 10 days by gavage from 7 weeks of age with a two-day interval in the middle [14–16].

**Table 1:** Data from rat mammary carcinogenesis experiments

No.	Study focus	Ref.	*n* ^a^	Birth cohort (date of birth)^b^	Parity	Fat in diet	Chemicals^c^	Radiation (age at exposure)	Termination
1	Chemicals	(14, 15)	417	1 (10 May 2002–17 Jan 2005)	No	High	None, MNU20/7, MNU40/7, PhIP	0 Gy; 0.2, 0.5, 1, 2 Gy (7 wk)	Lifetime^d^
2	Puberty	(16)	54	1 (18 Oct 2002–16 Jan 2003)	No	High	None MNU20/3, MNU20/7	2 Gy (3 wk) 0 Gy	Lifetime^d^
3	Miscellaneous	This study	159	1 (5 Dec 2002–3 Jan 2005) 1 (16 Jan 2003)	No No	Normal High	None MNU20/3, MNU20/7	0 Gy; 0.2, 0.5, 1, 2 Gy (7 wk) 2 Gy (3 wk)	Lifetime^d^
4	Carbon ions I	(17)	49	1 (23 Aug 2002–28 Nov 2003)	No	High	None	0 Gy	Lifetime^d^
5	Carbon ions II	(18)	570	1–2 (4 Oct 2004–16 Jul 2008)	No	Normal	None	0 Gy; 0.2, 0.5, 1, 2 Gy (1, 3, 7, 15 wk)^e^	90 wk
6	Strain	(19)	20	2 (9 Oct 2006–18 Jan 2007)	No	Normal	None	4 Gy (7 wk)	Lifetime
7	Neutrons	(20)	78	2–3 (1 Dec 2009–6 Oct 2011)	No	Normal	None	0 Gy	90 wk
8	Parity	(21)	111	3 (7 Jul 2011–10 May 2012)	Yes, no	Normal	None	0 Gy; 4 Gy (3 wk)	100 wk
9	Dose rate	(22)	196	3 (5 Nov 2010–8 Nov 2012)	No	Normal	None	0 Gy; 0.5, 1, 2, 3, 4 Gy (13 wk)	90 wk

wk, weeks of age.

^a^Number of rats.

^b^Birth cohorts are defined as follows: cohort 1 (born on 10 May 2002–14 July 2006, *n* = 900), cohort 2 (born on 9 October 2006–12 September 2010, *n* = 411), and cohort 3 (born on 21 October 2010–8 November 2012, *n* = 343).

^c^Numbers following abbreviation of chemical names are doses in mg/kg, numbers following the slash indicate age at exposure in weeks (see text for detail).

^d^Rats were sacrificed at 50 weeks of age if they had a palpable tumor; otherwise, they were sacrificed upon detection of the first tumor.

^e^Excluding data for 2 Gy exposure at 1 week of age due to premature cessation of the estrous cycle.

The experiment shown in number 3 of Table 1 is reported here for the first time. This experiment was conducted under approval by the Institutional Animal Care and Use Committee of the National Institute of Radiological Sciences (NIRS, approval number 17–1012). Female Jcl:SD rats were purchased from CLEA Japan and fed a standard CE-2 diet and sterile water ad libitum. Experiments were performed as described [14–16]. Briefly, rats were subjected to whole-body γ-irradiation (^137^Cs, 0.6 Gy/min) at 0, 0.2, 0.5, 1 or 2 Gy at 7 weeks of age. Another set of rats was irradiated at 2 Gy at 3 weeks of age and injected intraperitoneally with MNU (20 mg/kg) after 3 days (at 3 weeks of age) or at 7 weeks of age. These rats were fed a high-fat diet as described above after 9 weeks of age. Animals that showed signs of general deterioration or reached 50 weeks of age with one or more palpable tumors were euthanized and autopsied, animals found dead were also autopsied, and those without a palpable tumor at 50 weeks of age were sacrificed and autopsied upon later detection of the first tumor. During autopsy, palpable tumors were collected, fixed in 10% formalin, embedded in paraffin and processed for hematoxylin and eosin staining for histology [23]. The palpation record was used to determine the age at which tumors first developed.

### Statistical analysis

The first palpation of a mammary tumor that was later diagnosed as adenocarcinoma was treated as an incidence of mammary cancer, whereas any termination of observation before that was treated as censoring of observation. The attained age was categorized into 10-week terms, and the data were organized as the number of incidences in individual terms, the number of animal-weeks (as an equivalent of person-years in epidemiology) in the terms, and covariates (the mid-point of the attained age terms, radiation dose and age at exposure as continuous variables; fat, parity, chemical treatment and birth cohort as categorical variables). Birth cohorts were defined as follows: cohort 1 (born from 10 May 2002 to 14 July 2006, *n* = 900), cohort 2 (born from 9 October 2006 to 21 September 2010, *n* = 411) and cohort 3 (born from 21 October 2010 to 8 November 2012, *n* = 343), so that they included nearly equal numbers of rats without any treatment (*n* = 146, 139 and 138, respectively). Animals entered the analysis at the first week after birth if no treatment had been performed or at the first week after the end of the treatments otherwise.

The incidence rate of palpable mammary carcinoma *λ* (i.e. the number of cases per animal-week) was modeled as follows:


(1) \begin{equation*} \mathrm{ERR}\ \mathrm{model}:\lambda =A(t)\cdot \left(1+\sum{\beta}_i{I}_i\right)\cdot \left\{1+R\left(d,e,t\right)\right\}, \end{equation*}

(2) \begin{equation*} \mathrm{EAR}\ \mathrm{model}\ \left(\mathrm{standard}\right):\lambda =A(t)\cdot \left(1+\sum{\beta}_i{I}_i\right)+R\left(d,e,t\right), \end{equation*}

(3) \begin{equation*} \mathrm{EAR}\ \mathrm{model}\ \left(\mathrm{variant}\right):\lambda =A(t)\cdot \left\{1+\sum{\beta}_i{I}_i+R\left(d,e,t\right)\right\}, \end{equation*}

(4) \begin{eqnarray*} \mathrm{Generalized}\ \mathrm{Model}\ 1:\lambda =A(t)\cdot \left\{1+\sum{\beta}_i{I}_i+R\left(d,e,t\right)\right.\nonumber\\ \left.+\theta \cdot \sum{\beta}_i{I}_i\cdot R\left(d,e,t\right)\right\}, \end{eqnarray*}

(5) \begin{eqnarray*} \mathrm{Generalized}\ \mathrm{Model}\ 2:\lambda =A(t)\cdot \left[1+\sum{\beta}_i{I}_i+R\left(d,e,t\right)\right.\nonumber\\\left.+\sum{\theta}_i{\beta}_i{I}_i\cdot R\left(d,e,t\right)\right], \end{eqnarray*}

where *t* is attained age, *A*(*t*) is the baseline hazard function (for cohort 1 without exposure to any radiation or non-radiation factors), *i* is an indicator of non-radiation factors (where *i* = 1, 2, … and 8 represent high fat, parity, MNU20/3, MNU20/7, MNU40/7, PhIP, cohort 2 and cohort 3, respectively), Σ denotes sum for all *i*, *β_i_* is a coefficient for risk of non-radiation factor *i*, *I_i_* is a dummy variable for a non-radiation factor *i*, *d* is radiation dose, *e* is age at exposure, *R*(*d*, *e*, *t*) is the radiation-associated risk, *θ* is a coefficient describing the interaction between radiation and all non-radiation factors as an aggregate, and *θ_i_* is a coefficient describing the interaction between radiation and a non-radiation factor *i*. Herein, equation 4 is a special case of equation 5 in which all *θ_i_* have identical values, and equations 1 and 3 are special cases of equation 4 in which *θ* = 1 and 0, respectively. The interaction among non-radiation factors was modeled as additive herein; however, whether this interaction is additive or multiplicative does not matter because, in the experiments above, only one of the non-radiation factors was changed while the others were fixed.

The specific forms of *A*(*t*), *R*(*d*, *e*, *t*) and the set of *θ_i_* (***θ***) used herein were:


(6) \begin{equation*} A(t)=\exp \left({\alpha}_0+{\alpha}_1\ln \frac{t}{70}\right)\ \mathrm{or} \end{equation*}

(7) \begin{equation*} A(t)=\exp \left\{{\alpha}_0+{\alpha}_1\ln \frac{t}{70}+{\alpha}_2{\left(\ln \frac{t}{70}\right)}^2\right\}, \end{equation*}

(8) \begin{eqnarray*} R\left(d,e,t\right)={\gamma}_0d\cdot \exp \left\{{\gamma}_1\left(e-7\right){I}_{e<7}+{\gamma}_2\left(e-7\right){I}_{e\ge 7}\right.\nonumber\\\left.+{\gamma}_3\ln \frac{t}{70}\right\}\ \mathrm{or} \end{eqnarray*}

(9) \begin{eqnarray*} R\left(d,e,t\right)=\left({\gamma}_0d+{\gamma}_{\mathrm{Q}}{d}^2\right)\cdot \exp \left\{{\gamma}_1\left(e-7\right){I}_{e<7}\right.\nonumber\\\left.+{\gamma}_2\left(e-7\right){I}_{e\ge 7}+{\gamma}_3\ln \frac{t}{70}\right\}, \end{eqnarray*}

(10) \begin{equation*} \boldsymbol{\theta} =\left({\theta}_{\mathrm{fat}},{\theta}_{\mathrm{parity}},{\theta}_{\mathrm{chem}},{\theta}_{\mathrm{cohort}}\right) \end{equation*}

where *α*_0_, *α*_1_, *α*_2_, γ_0_, γ_1_, *γ*_2_, *γ*_3_ and *γ*_Q_ are constant values, *I* is an indicator which is 1 when the condition specified by the subscript is true and otherwise 0, and *θ*_fat_, *θ*_parity_, *θ*_chem_ and *θ*_cohort_ are interaction coefficients for high fat, parity, chemicals and birth cohorts, respectively (identical parameters were used for chemicals [*i* = 3–6] and cohorts [*i* = 7, 8]). Equations 2 and 3 are equivalent under these conditions, but the form of equation 3 allows comparison with the generalized models. Poisson regression, profile likelihood confidence intervals (CIs), Akaike’s information criterion and the likelihood ratio test were used for model fitting and comparison on R [24].

## RESULTS

### The non-radiation factors differentially impact baseline incidence

**Fig. 1:**
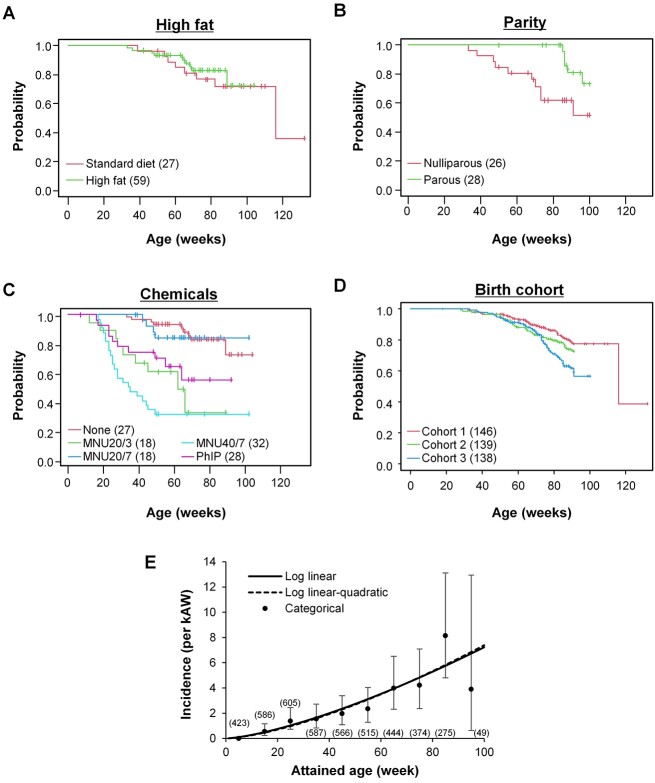
Baseline mammary cancer incidence in a pooled cohort of rats. (A–D) Kaplan–Meier plots depicting the influence of non-radiation factors that are indicated at the top of each plot. Numbers in parenthesis indicate the number of animals. (E) Baseline mammary cancer incidence by attained age. Log linear and log linear-quadratic models refer to equations 6 and 7 in the text. kAW, 10^3^ animal-weeks; MNU20/3, 20 mg/kg of MNU at 3 weeks of age; MNU20/7, 20 mg/kg of MNU at 7 weeks; MNU40/7, 40 mg/kg of MNU at 7 weeks. Numbers in parenthesis indicate animals at the beginning of each 10-week interval. Vertical bar, 95% CI.

To obtain insights into the modification of radiation-related mammary cancer incidence by age and various non-radiation factors, animal carcinogenesis data from past experiments were pooled and reanalyzed. The non-radiation factors in these experiments included high-fat diet, parity, treatment with chemical carcinogens and birth cohort. First, the impact of the non-radiation factors on cancer incidence in the absence of radiation was visualized with Kaplan–Meier plots (Fig. 1A–D) and quantified (Table 2). Herein, a log linear (equation 6) and log linear-quadratic function (equation 7) of attained age were tested. This analysis indicated a negligible effect of high-fat diet, a significant reduction by parity, a significant increase by chemical treatments except MNU20/7, and a significant influence of birth cohort 3 (Table 2). The addition of the log quadratic term for attained age did not improve the fit (Table 2, Fig. 1E). Thus, this analysis clarified the effect of the four factors on baseline incidence, and a log linear function of attained age was adopted for the baseline function in the subsequent analyses.

**Table 2:** Analysis of the baseline incidence: choice of attained age function and effects of non-radiation factors

Item	Attained age function	
	Log linear	Log linear-quadratic
Deviance	575.0^a^	574.9^a^
AIC	595.0	596.9
Parameters		
Constant^b^ (*α*_0_)	−5.4 [−5.8, −5.1]^***^	−5.4 [−5.8, −5.1]^***^
Log age/70 (*α*_1_)	1.4 [1.1, 1.8]^***^	1.5 [0.8, 2.1]^***^
(Log age/70)^2^ (*α*_2_)	—	0.0 [−0.4, 0.4]
High fat (*β*_1_)	0.1 [−0.5, 1.3]	0.1 [−0.5, 1.3]
Parity (*β*_2_)	−1.1 [−1.8, −0.1]^*^	−1.1 [−1.9, −0.1]^*^
MNU20/3 (*β*_3_)	6.8 [2.4, 15]^***^	6.9 [2.4, 15]^***^
MNU20/7 (*β*_4_)	0.4 [−1.1, 2.6]	0.4 [−1.1, 2.7]
MNU40/7 (*β*_5_)	13 [7, 23]^***^	13 [7, 23]^***^
PhIP (*β*_6_)	3.9 [1.2, 8.8]^**^	3.9 [1.2, 8.9]^**^
Cohort 2 (*β*_7_)	0.3 [−0.2, 1.3]	0.3 [−0.2, 1.3]
Cohort 3 (*β*_8_)	0.9 [0.2, 2.1]^*^	0.9 [0.2, 2.1]^*^

Data from all experiments (0 Gy, 614 animals; 42 611 animal-weeks) were analyzed. Brackets indicate 95% CIs; parentheses in brackets indicates that the value was not determinable (ND).

^a^ *P* = 0.63 by likelihood ratio test between the models.

^b^Natural logarithm of rate (per 10^3^ animal-week). AIC, Akaike’s information criterion; —, not applicable.

^*^ *P* < 0.05.

^**^ *P* < 0.01.

^***^ *P* < 0.001.

### Influence of age factors and shape of the dose response in the present data set

**Fig. 2:**
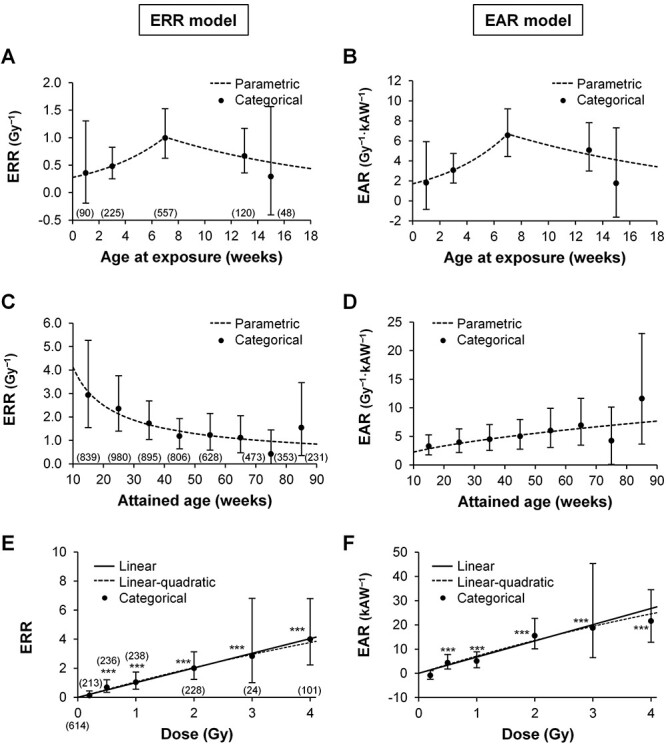
Influence of age and radiation dose on mammary cancer incidence in a pooled cohort of rats. EAR, excess absolute risk; ERR, excess relative risk; kAW, 10^3^ animal-weeks. (A–B) Influence of age at exposure on radiation-associated ERR per Gy (A) and EAR per Gy·kAW (B) at attained age 70 weeks. Numbers in parenthesis indicate animals in each group (common to A and B). (C–D) Influence of attained age on ERR per Gy (C) and EAR per Gy·kAW (D) associated with radiation exposure at age 7 weeks. Numbers in parenthesis indicate animals at the beginning of each 10-week interval (common to C and D). (E–F) Influence of radiation dose on ERR (E) and EAR (F) (age at exposure, 7 weeks; attained age, 70 weeks). Numbers in parenthesis indicate animals in each group (common to E and F), with the one under 0 indicating the number of nonirradiated animals. Vertical bar, 95% CI. ^*^^*^^*^P < 0.001 vs 0 Gy. The EAR model herein refers to both standard and variant EAR models as these are equivalent mathematically.

Age at exposure and attained age are important modifiers of radiation effects, and conventional ERR and EAR models already include flexibility regarding their interaction. ERR (equation 1) and standard EAR (equation 2) models were thus used to analyze the dependence of mammary cancer incidence on these age factors. Detailed results for the fit to equations are shown in Table 3 (see ‘ERR’ and ‘EAR [standard]’ columns), and representative predictions from the models are shown in Fig. 2. Herein, the categorical estimates of incidence showed a non-monotonic relationship with age at exposure (Fig. 2A–B). In both the ERR and EAR models, the age-at-exposure trend was better modeled as a log linear spline function with a knot at a peripubertal age of 7 weeks (equation 8) than as a simple log linear function (*P* = 0.003 and 0.002, respectively). This biphasic modeling indicated a significant increasing trend (before 7 weeks) of both ERR and EAR (Table 3, see *γ*_1_ values) and a near-significant decreasing trend (after 7 weeks) of ERR and EAR, respectively (Table 3, see *γ*_2_ values). Dependence of attained age was modeled as a log linear function of attained age (equation 8) based on the trend of categorical estimates (Fig. 2C–D). Therein, the decreasing trend in ERR and the increasing trend in EAR were significant (Table 3, see *γ*_3_ values). A log linear-quadratic function of log attained age in the ERR and EAR models did not significantly improve from a log linear function (*P* = 0.2 and *P* = 0.4, respectively). Thus, the effects of age at exposure and attained age were described with a log linear spline function with a knot at 7 weeks and a log linear function, respectively, as in equation 8.

**Table 3:** Analysis of the interactions between radiation and non-radiation factors

Item	Model				
	ERR	EAR (standard)	EAR (variant)	Generalized 1	Generalized 2
Deviance	2005.0^a^^,^^b^	2030.4	2030.4^c,d^	2004.6^a^^,^^c^^,^^e^	1995.3^b^^,^^d^^,e^
AIC	2033.0	2058.4	2058.4	2034.6	2031.3
Parameters					
Constant (*α*_0_)^f^	−5.4 [−5.7, −5.2]^***^	−5.7 [−6.0, −5.3]^***^	−5.7 [−6.0, −5.3]^***^	−5.5 [−5.8, −5.2]^***^	−5.5 [−5.9, −5.2]^***^
Attained age (*α*_1_)	1.3 [1.0, 1.6]^***^	1.0 [0.8, 1.3]^***^	1.1 [0.8, 1.3]^***^	1.3 [1.0, 1.6]^***^	1.3 [1.0, 1.6]^***^
High fat (*β*_1_)	0.6 [0.1, 1.4]^*^	0.4 [−0.3, 1.6]	0.4 [−0.3, 1.6]	0.6 [0.1, 1.5]^*^	0.07 [(>0), 0.8]^***^
Parity (*β*_2_)	−0.9 [(ND), −0.2]^*^	−1.4 [(ND), −0.3]^*^	−1.4 [(ND), −0.3]^*^	−1.0 [(ND), −0.3]^**^	−1.2 [(ND), (<0)]^*^
Chemical, MNU20/3 (*β*_3_)	3.4 [1.3, 6.6]^***^	7.0 [3.1, 13]^***^	7.0 [3.1, 13]^***^	3.9 [1.4, 8.1]^***^	4.9 [2.0, 9.9]^***^
Chemical, MNU20/7 (*β*_4_)	1.9 [0.9, 3.2]^***^	3.4 [1.5, 6.2]^***^	3.4 [1.5, 6.2]^***^	2.1 [0.9, 4.0]^***^	2.1 [0.7, 4.1]^**^
Chemical, MNU40/7 (*β*_5_)	8.5 [5.9, 12]^***^	17 [11, 26]^***^	17 [11, 26]^***^	9.6 [5.7, 16]^***^	12 [7.2, 20]^***^
Chemical, PhIP (*β*_6_)	2.3 [1.1, 3.8]^***^	4.8 [2.5, 8.3]^***^	4.8 [2.5, 8.3]^***^	2.6 [1.1, 4.9]^***^	3.1 [1.4, 5.8]^***^
Birth cohort 2 (*β*_7_)	0.1 [−0.2, 0.5]	0.3 [−0.1, 1.0]	0.3 [−0.1, 1.0]	0.1 [−0.2, 0.6]	0.3 [−0.1, 0.9]
Birth cohort 3 (*β*_8_)	0.9 [0.4, 1.6]^***^	1.3 [(<0), 2.5]	1.3 [(<0), 2.5]	1.0 [0.4, 1.9]^**^	1.1 [0.4, 2.2]^*^
Dose (Gy^−1^) (*γ*_0_)	1.0 [0.6, 1.5]^***^	6.7 [4.6, 9.3]^g^ ^***^	1.9 [1.1, 3.2]^***^	1.1 [0.6, 1.9]^***^	1.0 [0.5, 2.0]^***^
Age at exposure, <7 weeks (*γ*_1_)	0.18 [0.07, 0.32]^**^	0.19 [0.09, 0.32]^***^	0.19 [0.09, 0.32]^***^	0.18 [0.07, 0.31]^***^	0.18 [0.07, 0.32]^**^
Age at exposure, ≥7 weeks (*γ*_2_)	−0.08 [−0.18, 0.01]	−0.06 [−0.15, 0.01]	−0.06 [−0.15, 0.01]	−0.08 [−0.17, 0.01]	−0.03 [−0.17, 0.07]
Attained age (*γ*_3_)	−0.7 [−1.2, −0.2]^**^	0.6 [0.2, 0.9]^**^	−0.5 [−1.0, −0.001]^*^	−0.7 [−1.2, −0.2]^**^	−0.8 [−1.2, −0.3]^**^
Interaction, integrated (*θ*)	—	—	—	0.7 [0.3, 1.9]^***^	—
Interaction, high fat (*θ*_fat_)	—	—	—	—	30 [1.4, (ND)]^***^
Interaction, parity (*θ*_parity_)	—	—	—	—	0.3 [−1.0, (>2)]
Interaction, chemical (*θ*_chem_)	—	—	—	—	0.4 [0.06, 1.3]^*^
Interaction, birth cohort (*θ*_cohort_)	—	—	—	—	0.4 [−0.3, 4.4]

All cohorts from experiments 1–9 in Table 1 (1654 animals, 94 871 animal-weeks) were analyzed. Brackets indicate 95% CIs; parentheses in brackets indicates that the value was not determinable (ND) or determined as a range as indicated because the optimization calculation did not converge. AIC, Akaike’s information criterion; —, not applicable.

^a^ *P* = 0.5.

^b,e^ *P* = 0.002.

^c,d^ *P* < 0.001 between models with the same superscript letters (*a*–*e*) by likelihood ratio test.

^f^Natural logarithm of rate (per 10^3^ animal-week).

^g^Per 10^3^ animal-week.

^*^ *P* < 0.05.

^**^ *P* < 0.01.

^***^ *P* < 0.001.

The shape of the dose response was modeled as linear or linear-quadratic in the ERR and standard EAR models. Having hundreds of animals per dose group, the categorical estimates were significant at doses ≥0.5 Gy, but not at 0.2 Gy (*P* = 0.33), compared with 0 Gy (Fig. 2E–F). These estimates exhibited an apparently linear relationship with dose (Fig. 2E–F), and addition of the quadratic term did not significantly improve the fit from linearity in either the ERR or EAR model (*P* = 0.6 for both models). Thus, a linear function was used for the dose response hereafter.

### Heterogeneity exists in interactions between radiation and various non-radiation factors

**Fig. 3:**
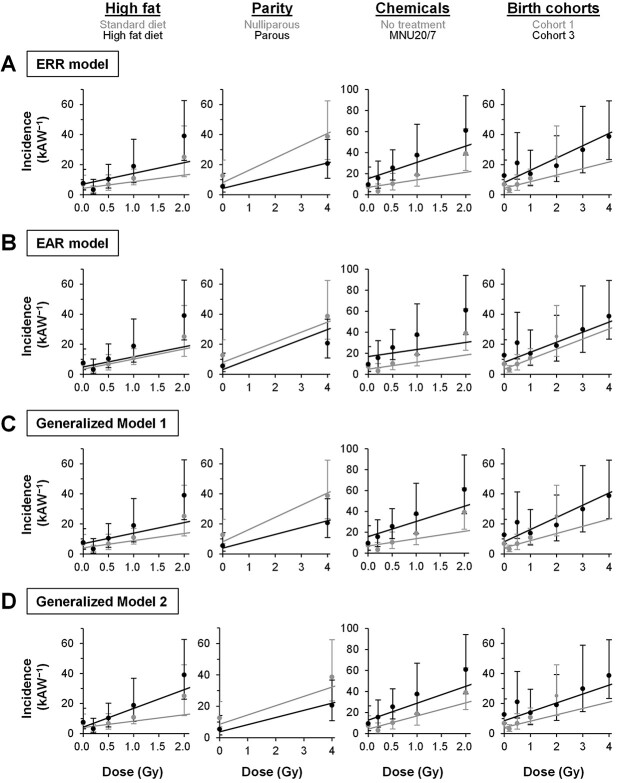
Comparison of four models on the interaction of non-radiation factors with radiation on mammary cancer incidence in a pooled cohort of rats. Dots and vertical bars are shown commonly for all models and indicate categorical estimates and their 95% CIs, respectively, calculated without assuming dose dependence or interactions. In A–D, a single model (boxed) was used to analyze all data, and the results are shown in grey and black lines for representative groups of the individual four categories of non-radiation factors (shown at the top). The EAR model herein refers to both standard and variant EAR models as these are equivalent mathematically. kAW, 10^3^ animal-weeks; MNU20/7, 20 mg/kg of MNU at age 7 weeks.

We next set out to elucidate the interaction of non-radiation factors with radiation. For this purpose, the data were analyzed using Generalized Models 1 and 2 (equations 4 and 5). The variant EAR model (equation 3), nested in Generalized Models 1 and 2 and equivalent to the standard EAR model, was used only to make statistical comparisons between models. In Generalized Model 1, the optimal *θ* value determined by fitting indicates the magnitude of the interaction between radiation and the non-radiation factors (i.e. *θ* < 0, antagonistic; *θ* = 0, additive; 0 < *θ* < 1, supra-additive and sub-multiplicative; *θ* = 1, multiplicative; *θ* > 1, supra-multiplicative); the same applies to *θ_i_* in Generalized Model 2. The details of fitting results are shown in Table 3 (see ‘EAR [variant]’, ‘Generalized 1’ and ‘Generalized 2’), and categorical estimates for representative groups and example predictions from the models are illustrated in Fig. 3. As a result, Generalized Model 1, which treats all non-radiation factors as an aggregate, significantly (*P* < 0.001) improved from the variant EAR model (a model assuming additivity) but not from the ERR model (which assumes multiplicativity) (Table 3). Generalized Model 1 gave a *θ* value of 0.7 (95% CI [0.3, 1.9]), which suggested a significant departure from additivity and supported a multiplicative interaction (Table 3). Examples shown in Fig. 3 illustrate the general trend of good fit of the ERR model and Generalized Model 1 compared with the EAR model.

Of note, Generalized Model 2, which treats the four non-radiation factors as having different interactions, was significantly improved from the ERR model, variant EAR model and Generalized Model 1 (*P* = 0.002 or *P* < 0.001) (Table 3). This model gave a *θ*_fat_ value of 30 (lower limit of 95% CI, 1.4), which is significantly larger than 1 and hence indicated a supra-multiplicative interaction between radiation and high-fat diet. Regarding parity, this model gave a *θ*_parity_ value of 0.3, with its 95% CI covering both 0 and 1, indicating that the interaction could not be determined as either additive or multiplicative. The *θ*_chem_ value was 0.4 (95% CI [0.06, 1.3]), which significantly departed from 0 and supported a multiplicative interaction between radiation and chemical treatments. Regarding the birth cohorts, the model yielded a *θ*_cohort_ value of 0.4 (95% CI [−0.3, 4.4]), indicating an interaction that could not be determined as either additive or multiplicative. Examples in Fig. 3 illustrate the better fit of Generalized Model 2 than the other three models. In addition, use of generalized models did not overtly change the parameter estimates from the ERR and EAR models (Table 3).

Thus, the analysis indicated that the interactions of these four factors with radiation are diverse and should hence be treated separately for the model to better describe the data. Further, when these factors were treated as one entity, the results showed a multiplicative interaction with radiation.

## DISCUSSION

In the present study, the pooled incidence data from past experiments (involving 1654 rats) were reanalyzed to quantify: (1) the modification by age at exposure and attained age; (2) the shape of the dose response; and (3) the interaction of the effects of high-fat diet, parity, chemicals and birth cohorts with radiation. Regarding the age effects, the present analysis supports a peripubertal peak in susceptibility as well as a change associated with attained age, both of which are concordant with a previous report on the LSS cohort [7]. The dose response exhibited linearity, but the incidence in the lowest dose group (0.2 Gy, *n* = 213 rats) did not differ significantly from the baseline incidence (*n* = 614 rats). Importantly, there was significant heterogeneity—ranging from additivity to supra-multiplicativity—in the modification of radiation-associated risk by non-radiation factors.

Compared with recent findings in epidemiology, the present results support their main conclusions and provide a guide for future research. First, regarding the effect modification by age at exposure, the recent LSS study suggested a non-monotonic trend with a knot around the pubertal period [7], which is supported by the present study. Our analysis showed a peak of ERR and EAR around puberty, which coincides with the results of ERR, rather than EAR, in the LSS cohort [7]. Regarding the modification by attained age, the previous two studies on the LSS cohort support a significant decreasing trend in ERR [2, 7]. The curve for the increase of EAR with attained age was convex upward in both studies. The contribution of the log quadratic term of attained age to the modification of EAR, suggested in the LSS cohort [7], was not reproduced in the present rat cohort; this may reflect the difference in the attained-age dependence of the baseline incidence between the cohorts. Second, regarding the dose response, both studies indicate linear relationships, with no significant departure from linearity. The present study supports a strong linearity up to a high dose of 4 Gy, although the linearity was attenuated over 2 Gy in the LSS cohort [7]. In this sense, the prominent dose rate effect observed in the rats [22] cannot be explained by the curvature of dose response, as the current radiological protection system assumes [25]. Third, regarding the effect modification by non-radiation factors, the LSS study found no significant modification of ERR by body mass index, parity or smoking [7], indicating no significant departure from simple multiplicativity. In contrast, the present study suggests the possibility of more diverse interactions by similar non-radiation factors, i.e. dietary fat, parity and carcinogenic chemicals. Relatively small variations in such factors may exist within the Japanese female population of the LSS cohort, suggesting the need for a pooled analysis of cohorts including non-Japanese populations with information about lifestyle factors. The birth cohort in the LSS and the present studies may have different meanings, as that in the former may represent the rapid Westernization of lifestyles in Japan whereas the latter may include genetic fluctuation (note that Jcl:SD is an outbred closed colony strain), changes in breeding conditions, difference in the palpation skill and variability of the natural components in diet.

The heterogeneity in the interaction of various factors suggests diversity in their mechanisms of interaction. The peripubertal peak of the age-at-exposure dependence suggests its association with the very rapid cell proliferation that occurs during puberty [26]. The genome of radiation-induced rat mammary cancer frequently displays large structural abnormalities [27–29], suggesting the influence of error-prone end-joining machinery. The effect of *Brca1* knockout on radiation-induced mammary carcinogenesis was minimal in rats irradiated immediately after, but not before, puberty [30]. These findings imply that the error-free DNA repair by homologous recombination is minimal during puberty, allowing genesis of structural abnormalities by error-prone repair mechanisms. Regarding the interaction with non-radiation factors, it is generally known from biologically based mathematical models that two factors acting on the initiation step of carcinogenesis tend to interact additively, those acting separately on the initiation and promotion/progression steps display multiplicative interactions and those acting on promotion/progression steps show variable interactions [31–33]. The supra-multiplicative modification by dietary fat may thus involve its action on the promotion/progression phase, which could include (i) upregulation of estrogen production via adipocyte aromatase, (ii) elevation of cell proliferation via inflammatory adipokines, and (iii) elevation of protein synthesis and cell proliferation via circulating insulin and leptin [34–36]. The multiplicative interaction of mutagenic chemicals (MNU and PhIP) is odd given that they may act on the initiation step—as would radiation. Nevertheless, previous studies have suggested promotion-like effects of radiation on cells harboring chemically induced mutations [14, 15, 37]. Reduction of such supra-multiplicative and multiplicative interactions would establish a path for deliberately and retrospectively controlling cancer risks from known radiation exposure in the past [38], as these interactions were observed in situations where dietary fat or chemicals were applied subsequent to radiation exposure.

Limitation of the present study may include the insufficient number of animals, resulting in a lack of statistical power for identifying the interaction between radiation and other factors.

Taken together, the present reanalysis of archival rat mammary carcinogenesis data supports major conclusions from epidemiology on the dose response and effect modification by age. It also suggests significant heterogeneity in the interactions of non-radiation factors with radiation. Modeling of the interaction between radiation and non-radiation factors did not affect the shape of dose response or age effects, but its potential impact would be substantial for risk estimation in populations exposed to factors that exhibit supra-multiplicative interactions. Clarification of the effect modification by non-radiation factors will contribute to improvement in risk transfer from epidemiology of atomic bomb survivors to populations worldwide [39] and consideration of radiation risk in the context of overall environmental exposures (or the ‘exposome’) [40]. Further analyses of other archival data—especially on cancer risk of other tissues—are thus warranted.
